# The current status and factors related to the preparation of home first-aid kits in China

**DOI:** 10.3389/fpubh.2022.1036299

**Published:** 2022-11-28

**Authors:** Pu Ge, Jinzi Zhang, Ke Lyu, Yuyao Niu, Qiyu Li, Ping Xiong, Jiaxin Liu, Yajie Yang, Yuqian Deng, Xialei Li, Wenli Yu, Mei Yin, Xinying Sun, Xu Han, Yibo Wu

**Affiliations:** ^1^Institute of Chinese Medical Sciences, University of Macau, Macao, China; ^2^School of Humanities and Social Sciences, Harbin Medical University, Harbin, China; ^3^China Medical University, Shenyang, China; ^4^Department of English, Faculty of Arts and Humanities, University of Macau, Macao, China; ^5^School of Humanities and Management, Jinzhou Medical University, Jinzhou, China; ^6^Xiangya School of Nursing, Central South University, Changsha, China; ^7^School of Nursing, Peking University, Beijing, China; ^8^School of Pharmaceutical Sciences, Shandong University, Jinan, China; ^9^School of Foreign Languages, Weifang University of Science and Technology, Weifang, China; ^10^School of Public Health, Peking University, Beijing, China; ^11^School of Marxism, Liaoning University, Shenyang, China

**Keywords:** self-efficacy, health literacy, home first-aid kit, cross-sectional study, Emergency Management, Big Five personality

## Abstract

**Background:**

Home first-aid kits can play an important role when residents are injured, suddenly become seriously ill or suffer from disasters.

**Purpose:**

To explore the home first-aid kit preparedness of Chinese residents and the relationship between demographic sociological characteristics, self-efficacy, Big Five personality, health literacy and home first-aid kit preparation behavior.

**Methods:**

A cross-sectional study was conducted. Information was collected through a self-designed questionnaire consisting of sociological characteristics, the New General Self-Efficacy Scale (NGSES), the Health Literacy Scale Short-Form (HLS-SF12), and the 10-item short version of Big Five Inventory (BFI-10). Rank sum test, Chi-square test, and logistic regression were used to explore the relationship between independent variables and home first-aid kit preparation behavior.

**Results:**

A total of 9,344 respondents were included, and 2,156 (23.07%) prepared home first-aid kits. Among the respondents who had prepared their home first-aid kits, disinfection supplies (85.20%), medical masks (84.51%), commonly used drugs (82.79%) were the most frequently available. The respondents whose geographic area was Central and Western China, permanent residence in the urban area, secondary education or above, monthly income of 3,000 RMB or above, health care cost-bearing method mainly resident health insurance, high subgroup of self-efficacy, high subgroup of health care dimension in health literacy, and whose openness and conscientiousness was high subgroup were more likely to prepare home first-aid kits (*P* < 0.05).

**Conclusion:**

The preparation rate for home first-aid kits in China is less than a quarter. The public's home first-aid kit preparation behavior is related to demographic characteristics, self-efficacy, health literacy, and the openness of the Big Five personality. A multi-level linked national emergency literacy education system should be established to enhance the residents' attention to home first-aid kits and improve the residents' ability to prevent emergencies.

## Introduction

A first-aid kit is a container with first-aid medicines or medical equipment, which can be classified as home first-aid kits, outdoor first-aid kits, vehicle first-aid kits, etc. The home first-aid kit is a comprehensive first-aid kit for accidents or disasters such as knife cuts or earthquakes. It is one of the important guarantees for the life safety of family members. It can play an important role when a family member is injured, suffers a sudden emergency illness, or encounters a disaster.

In Japan, first aid kits can be found everywhere in the living, studying, and working environment (1). U.S. public health agencies have long worked to help people cope with disasters and emergencies of all kinds. In 2003, the U.S. established a specialized agency, the Federal Emergency Management Agency (FEMA), which provides knowledge and corresponding funding support for state and local government emergency preparedness efforts (2). The agency had launched a national emergency preparedness campaign in the United States to encourage the public to prepare emergency items such as home first-aid kits. However, a study showed that only 30%−40% of Americans had emergency items such as home first aid kits (2, 3). The British government had distributed a handbook to every household called “Preparing for Emergencies: What You Need to Know.” Page et al. surveyed the British public's use of the handbook after the London bombings and found that <50% of the London public had read it (4, 5). At the same time, they found that after the London bombings, the London public increased its emergency preparedness, such as home first aid kits. In Australia, about one in five households are adequately prepared with emergency items such as cell phones, first aid kits, batteries, etc (6).

China is a populous country with a high frequency of natural disasters such as earthquakes, typhoons, and floods. On May 12, 2008, an 8.0 magnitude earthquake struck Wenchuan, Sichuan, China, causing nearly 70,000 deaths and nearly 400,000 injuries; On April 14, 2010, a 7.1 magnitude earthquake struck Yushu, Qinghai, China, killing more than 2,000 people and injuring tens of thousands. On average, 5.8 flood disasters and 6.9 typhoons occur in China each year, but studies show that the preparedness of Chinese residents' home first-aid kits is not optimistic (6). In 2018, the Ministry of Emergency Management of PRC was established, and in 2019, to improve the ability of disaster prevention and emergency rescue capability and protect people's lives and properties, the Ministry of Health issued the Standardized Management Measures for Emergency Management Standardization (7). In terms of emergency preparedness, although the Chinese government has issued relevant policies and some people have prepared first-aid kits, the preparation of home first-aid kits for Chinese citizens is not optimistic.

At present, there are relatively more studies on residents' emergency preparedness behaviors, but relatively few studies on residents' home first-aid kit preparedness. Some characteristics and behaviors of the public may affect the preparation of family first-aid kits. According to the cognitive theory, self-efficacy will affect the public's behavior motivation, and it is an important factor to decide the individual's behavior (8). Self-efficacy refers to an individual's subjective judgment on whether he can successfully reach a certain level or cope with a certain dilemma. Even if people know that a certain behavior will lead to good results, they may not engage in this behavior, but first, judge and evaluate whether they can implement this behavior. This process is the performance of self-efficacy (9). A study by Ning et al. found that personal emergency preparedness behavior was related to emergency knowledge, attitude, risk perception, self-efficacy, and participation in related educational activities (2). A study by Hamann et al. (10) found that self-efficacy and response efficacy was positively correlated with home emergency preparedness behaviors. The World Health Organization defines health literacy as the ability to make effective health decisions to improve human health (11). Sørensen et al. (12) developed a comprehensive definition of health literacy, including the knowledge, motivation, and ability to acquire, understand, evaluate, and apply information in everyday life to make judgments and decisions in healthcare, disease prevention, and health behaviors, to maintain and improve life quality throughout the whole lifetime. Health literacy is an important factor related to the use of medical services. Lack of health literacy has a great influence on all kinds of health behaviors and outcomes, including low utilization rate of preventive measures and emergency services, high hospitalization rate, and high medical expenses (13). Personality represents a series of thinking patterns and habitual behaviors within an individual and influences the individual's response to external stimuli and interactions with others in society (14). Personality, especially the Big Five personality, is closely related to people's behavior and results. The five factors of personality are nervousness, extroversion, openness, agreeableness, and conscientiousness. Among them, people with high nervousness have poor emotional stability and are prone to anxiety, depression, or impulsiveness. Extroverted people are energetic, extroverted and sociable. Open people are creative, full of curiosity and more willing to accept new ideas. Agreeableness reflects human trustworthiness, altruism and compassion. Conscientiousness embodies self-discipline, motivation and the ability to achieve responsibility (i.e., self-discipline, due diligence, and consideration) (15). A study investigated the relationship between COVID-19's personality and hoarding materials in an emergency and found that individuals with high agreeableness, neuroticism, and openness tend to hoard materials in an emergency (16). Based on the above theory and research, we believe that there is a correlation between residents' demographic sociological characteristics, self-efficacy, health literacy, personality, and first aid kit preparation.

This study aims to investigate the preparedness of home first-aid kits among Chinese residents and the related factors of first-aid kit preparation. Specifically, we explored respondents' home first-aid kit preparation rate, emergency supplies in home first-aid kits, and the relationship between respondents' demographic characteristics, self-efficacy, health literacy, Big Five personality, and home first-aid kit preparation behavior.

## Materials and methods

### Study design

The study was conducted in mainland China from July 10^th^, 2021, to September 15^th^, 2021 using a multi-stage sampling method. Four municipalities, Beijing, Tianjin, Shanghai, and Chongqing, and the capitals of 23 provinces and five autonomous regions across China were directly selected for sampling, and 2–6 cities were selected using the random number table method in the non-capital prefecture-level administrative regions of each province and each autonomous region to ensure that the ratio of the number of cities selected in Eastern China, Central China and Western China is close to the ratio of the number of cities in Eastern China, Central China and Western China. A total of 120 cities were selected for sampling at last. Based on the data report of the “Seventh National Census in 2021” (17), quota sampling was conducted to classify the quota attributes into gender, age, and urban-rural distribution. The investigators or survey teams (≤10 people) were recruited openly and trained in the sample cities. At least one investigator or one survey team was recruited in each city, with each investigator responsible for collecting 30–90 questionnaires, and each team responsible for collecting 100–200 questionnaires.

The survey was carried out through an online questionnaire platform called Wenjuanxing, the most popular survey software in China (https://www.wjx.cn/), by investigators issuing questionnaires to residents one-on-one. The participants signed the informed consent form and answered the questionnaires by clicking on the link, and the investigators input the questionnaire number. If the respondent could think but did not have sufficient action ability to answer the questionnaire, the investigator would conduct a one-on-one interview to help the respondents answer the questions.

### Participants

#### Inclusion criteria

(1) Age >18; (2) Nationality of the People's Republic of China; (3) Permanent resident population in China with an annual travel time ≤1 month; (4) Voluntary participation in the study and completion of the informed consent form; (5) Participants can complete the web-based questionnaire by themselves or with the help of investigators; (6) Participants can understand the meaning of each item in the questionnaire.

#### Exclusion criteria

**Figure 1:**
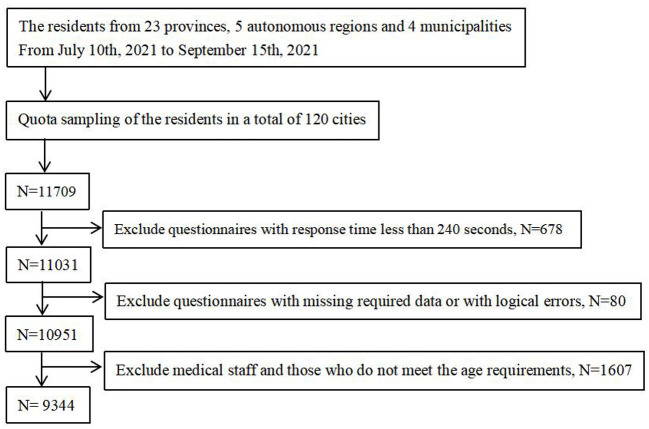
Flowchart of participants' enrollment.

(1) Persons with unconsciousness or mental disorders; (2) Persons involved in other similar research projects. (3) Medical staff. Initially, 11,709 participants from 120 cities in mainland China finished the questionnaire. After excluding questionnaires that took <240 s to answer and those that did not meet the requirements of this study 9,344 residents were enrolled in this study. Figure 1 shows a detailed flowchart of the enrollment. The effective rate of the investigation reached 79.80%.

### Instruments

The questionnaire consists of three parts focusing on the current status of residents' home first-aid kit preparation behavior and related factors. The first part investigated the social-demographic characteristics of the residents, which included gender, age, province, permanent residence (urban, rural), education level, per capita monthly income of the family, the current main way of bearing medical expenses, etc. The second part investigated the current status of residents' home first-aid kit preparation behavior, including two entries (one single-choice entry, and one multiple-choice entry). The third part is a series of standard scales, including the 10-item short version of the Big Five Inventory (BFI-10), the Short-Form Health Literacy Instrument (HLS-SF12), and the New General Self-Efficacy Scale (NGSES).

#### Items for residents' home first-aid kit preparation behavior

This section includes two entries (one single-choice entry, and one multiple-choice entry). The entries in this part were designed based on market research on the current first-aid kit, a review of relevant literature, and expert consultation (18–24).

The first entry in this part is *Does your household currently has a first-aid kit?* Respondents who answered “No” to this entry did not need to answer the next entry.

The second entry in this part is *What emergency supplies are in the home first-aid kit of your household?* There are six options in this entry, which are disinfection items (such as iodine), dressing supplies (such as gauze, and bandages), commonly used drugs (such as non-steroidal anti-inflammatory drugs, antidiarrheal drugs), special drugs (such as Suxiaojiuxin pills—traditional Chinese medicine, emergency medicine for angina pectoris) and other items. Respondents who choose “Other Items” need to fill in the name of the items. The order in which the options appear in the multiple-choice entry is random for each respondent.

#### The 10-item short version of the Big Five Inventory

Big Five Inventory (BFI-10) was applied to measure the personality characteristics of residents, including Extraversion, Agreeableness, Conscientiousness, Neuroticism, and Openness, on a 5-point Likert-type scale ranging from 1 (totally disagree) to 5 (totally agree) (25, 26). The scores of Extraversion were summed of the scores of item 1R and item 6, the scores of Agreeableness were combined with the scores of item 2 and 7R, Conscientiousness as 3R and 8, Neuroticism as 4R+9, and Openness as 5R+10 (R = item is reversed-scored). Several studies have shown that BFI-10 has good reliability and validity (27–29). In a previous study, the reliability levels of the BFI-10 proved satisfactory using Cronbach's α analysis: Extraversion (α = 0.723), Agreeableness (α = 0.759), Conscientiousness (α = 0.786), Neuroticism (α = 0.753) and Openness to experience (α = 0.714) (30). The higher the respondent's personality trait score, the more significant the respondent's personality trait.

#### The Health Literacy Scale Short-Form

The health literacy of the respondents was measured by Health Literacy Scale Short-Form (HLS-SF12) (31). The scale includes three dimensions of health care, disease prevention, and health promotion, with a total of 12 items, and each item is scored on a 4-point scale (1 = very difficult, 2 = difficult, 3 = easy, 4 = very easy). A standardized HL index was calculated using a formula with an index range of 0–50, and its score was positively correlated with the health literacy of the respondents. The calculation formula is, index = (mean – 1) ^*^ (50/3), where the mean is the average of all items involved in each individual, 1 is the minimum possible value of the mean (when the minimum value of the index is 0), 3 is the range of the mean, and 50 is the maximum value of the index. The higher the index, the higher the health literacy level of the investigation respondents. In the study, Cronbach's coefficient of the scale was 0.940, and Cronbach's coefficients of the three subscales of health care, disease prevention, and health promotion were 0.856, 0.860, and 0.868, respectively, with good reliability. In this study, the health literacy of the investigation respondents was divided into the high group (over 33 points) and the low group (33 points and below) about relevant literature.

#### New General Self-Efficacy Scale

The New General Self-Efficacy Scale (NGSES) was used to measure the respondents' self-efficacy level. It consists of 8 items and each item was scored on a five-point Likert scale (1 = completely disagree, 2 = disagree, 3 = neutral, 4 = agree, 5 = strongly agree) (32). All items were scored positively, and the total score on the scale was calculated by summing all item's scores. The total score of the scale ranges from 8 to 40 points. The higher the score, the higher the self-efficacy level of the respondents. The Cronbach's coefficient of the scale in this study was 0.943, which means that the scale had good reliability in this study.

### Statistical methods

Data entry and analysis were performed using SPSS™ for Windows (version 27.0; SPSS Inc., Chicago, IL, USA). The quantity and percentage of categorical variables were calculated using descriptive statistics. Scale scores were tested for normality. For normally distributed data, the mean and standard deviation were used for statistical description, and non-normally distributed data, the median and interquartile range were used for statistical description. Regarding the relevant literature, all scale scores were converted into dichotomous variables (high grouping and low grouping). The independent variables in the study include the demographic and sociological characteristics, health literacy, personality, and self-efficacy of the respondents. The dependent variable is whether the respondents prepare home first-aid kits. The Chi-square test and rank sum test was used for univariate analysis, and multivariate binary stepwise logistic regression was used for multivariate analysis. The inclusion and exclusion criteria of variables were: *P* = 0.05 and *P* = 0.10, respectively. Unless otherwise stated, the test level of statistical tests was α = 0.05.

### Quality control

The study conducted two rounds of pre-investigation and two rounds of expert consultation before the formal survey. Trained investigators distributed questionnaires to respondents and registered their codes one-on-one and face-to-face. Every Sunday evening during the investigation process, members of the research group communicated with the investigators to summarize, evaluate and give feedback on the questionnaires they collected. After the questionnaires were collected, two people conducted back-to-back logic checks and data screening. If singular values are found during data analysis, the original questionnaire must be found and checked with the investigator before proceeding to the next step of the analysis.

## Research results

### Common method bias

Common method bias shows that Harman's single-factor method showed five factors with eigenvalues >1, and the variance contribution rate of the first main factor was 34.32%, not exceeding 40%, indicating that there was no common method bias.

### Basic information of respondents and preparation of home first-aid kit

**Figure 2:**
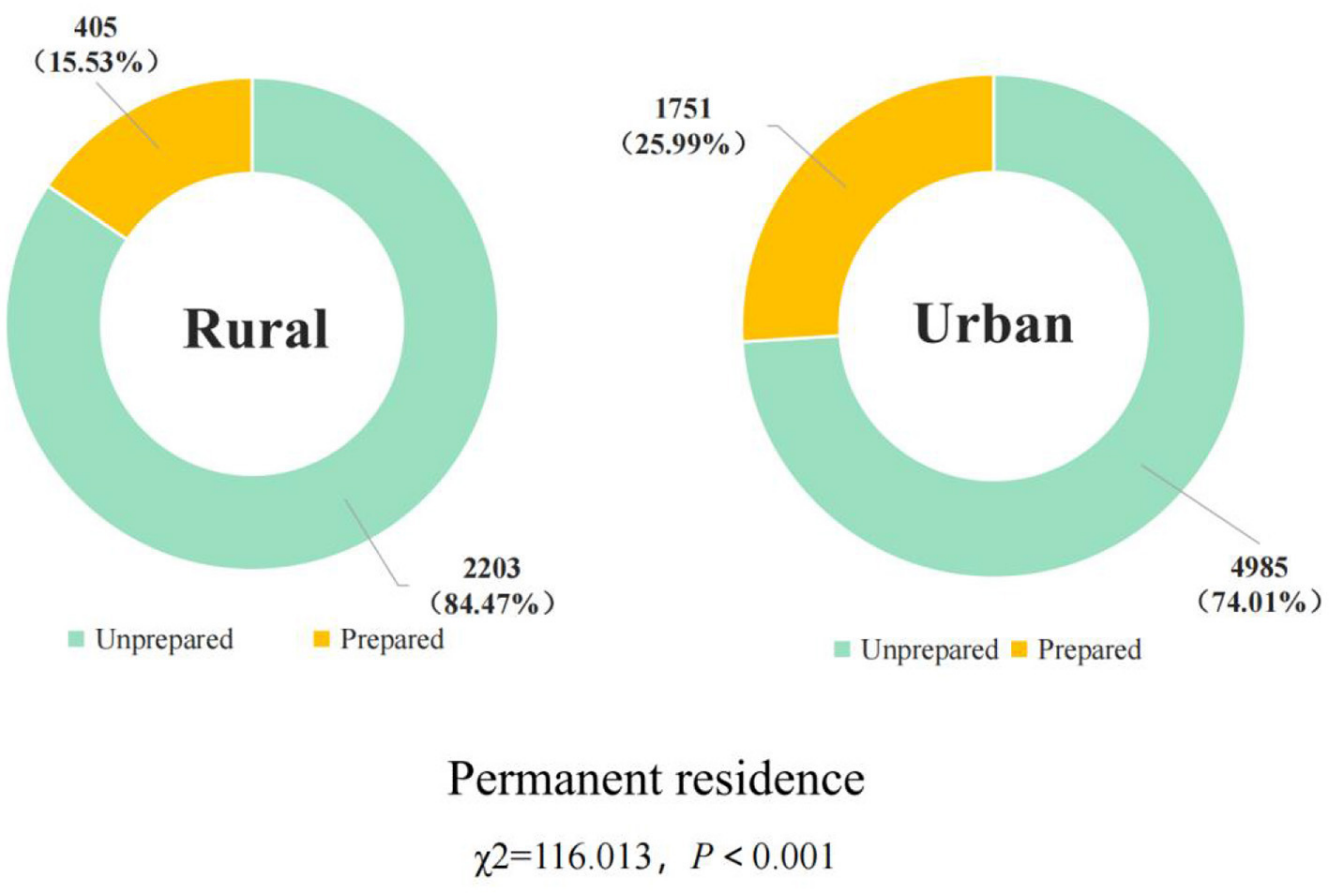
Preparation of home first-aid kit by permanent residence.

**Figure 3:**
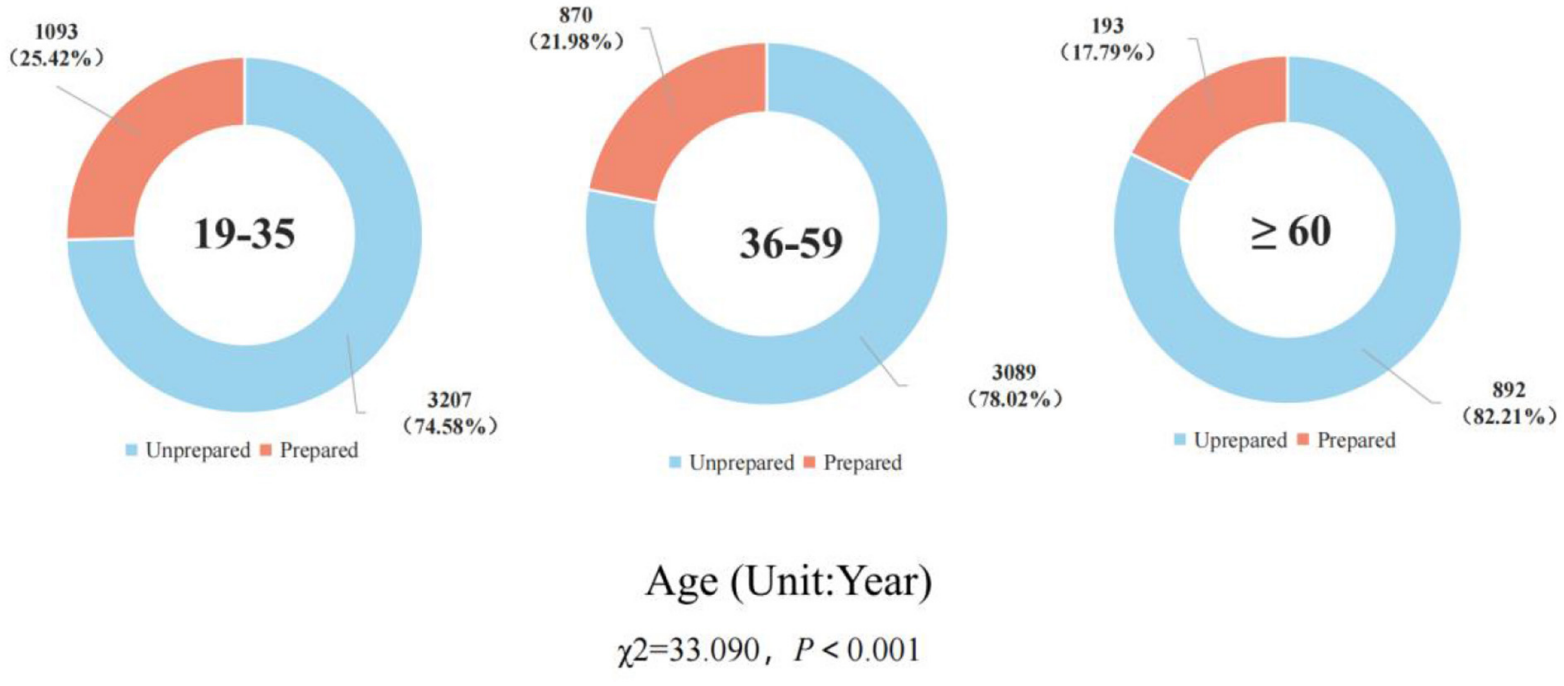
Preparation of home first-aid kit by different age groups.

A total of 9,344 valid questionnaires were recovered, with an effective rate of 84.71%. A total of 2,156 households in the study had home first-aid kits, and the home first-aid kit preparation rate was 23.07%. The assignment status of the independent and dependent variables is shown in Supplementary Table 1. There were statistically significant differences in the preparation rate in terms of permanent residence, age, highest educational level, per capita monthly household income, and the main way of bearing medical expenses by Chi-square test (*P* < 0.001, Table 1). Figures 2, 3 shows the preparation status of home first-aid kits by permanent residence and different age groups. The preparation status of home first-aid kits by gender and region can be seen in Supplementary Figures 1, 2.

**Table 1:** Basic information of respondents and preparation status of home first-aid kit.

**Categorical variables**	**Preparation status of home first-aid kit**			***χ^2^***	***P*-values**
	**Overall (percentage)**	**Number of unprepared (percentage)**	**Number of prepared (percentage)**		
**Total people**	9,344 (100%)	7,188 (76.93%)	2,156 (23.07%)	—	—
**Gender**				0.249	0.618
Male	4,342 (46.47%)	3,330 (76.69%)	1,012 (23.31%)		
Female	5,002 (53.53%)	3,858 (77.13%)	1,144 (22.87%)		
**Region**				2.226	0.329
Eastern	4,765 (51.00%)	3,694 (77.52%)	1,071 (22.48%)		
Central	2,408 (25.77%)	1,830 (76.00%)	578 (24.00%)		
Western	2,171 (23.23%)	1,664 (76.65%)	507 (23.35%)		
**Permanent residence**				116.013	<0.001
Rural	2,608 (27.91%)	2,203 (84.47%)	405 (15.53%)		
Urban	6,736 (72.09%)	4,985 (74.01%)	1,751 (25.99%)		
**Age (year)**				33.090	<0.001
19–35	4,300 (46.02%)	3,207 (74.58%)	1,093 (25.42%)		
36–59	3,959 (42.37%)	3,089 (78.02%)	870 (21.98%)		
≥60	1,085 (11.61%)	892 (82.21%)	193 (17.79%)		
**Highest educational level**				118.630	<0.001
Primary school and below	989 (10.58%)	878 (88.78%)	111 (11.22%)		
Middle School (including junior high school/high school/secondary school)	2,727 (29.18%)	2,139 (78.44%)	588 (21.56%)		
Junior college and undergraduate	5,006 (53.57%)	3,744 (74.79%)	1,262 (25.21%)		
Graduate (including Master's and Ph.D. students)	622 (6.66%)	427 (68.65%)	195 (31.35%)		
**Per capita monthly household income, yuan**				121.622	<0.001
≤3,000	2,755 (29.48%)	2,294 (83.27%)	461 (16.73%)		
3,001–6,000	3,656 (39.13%)	2,807 (76.78%)	849 (23.22%)		
6,001–9,000	1,547 (16.56%)	1,124 (72.66%)	423 (27.34%)		
>9,000	1,386 (14.83%)	963 (69.48%)	423 (30.52%)		
**The main way of bearing medical expenses**				32.274	<0.001
Medical insurance for residents (including medical insurance for urban residents and the new rural cooperative medical care)	4,502 (48.18%)	3,504 (77.83%)	998 (22.17%)		
Self-paying	1,864 (19.95%)	1,494 (80.15%)	370 (19.85%)		
Employee medical insurance,	2,595 (27.77%)	1,907 (73.49%)	688 (26.51%)		
Other types (including commercial medical insurance, and public medical care)	383 (4.10%)	283 (73.89%)	100 (26.11%)		

### Storage of items in home first-aid kits for respondents who have prepared

**Figure 4:**
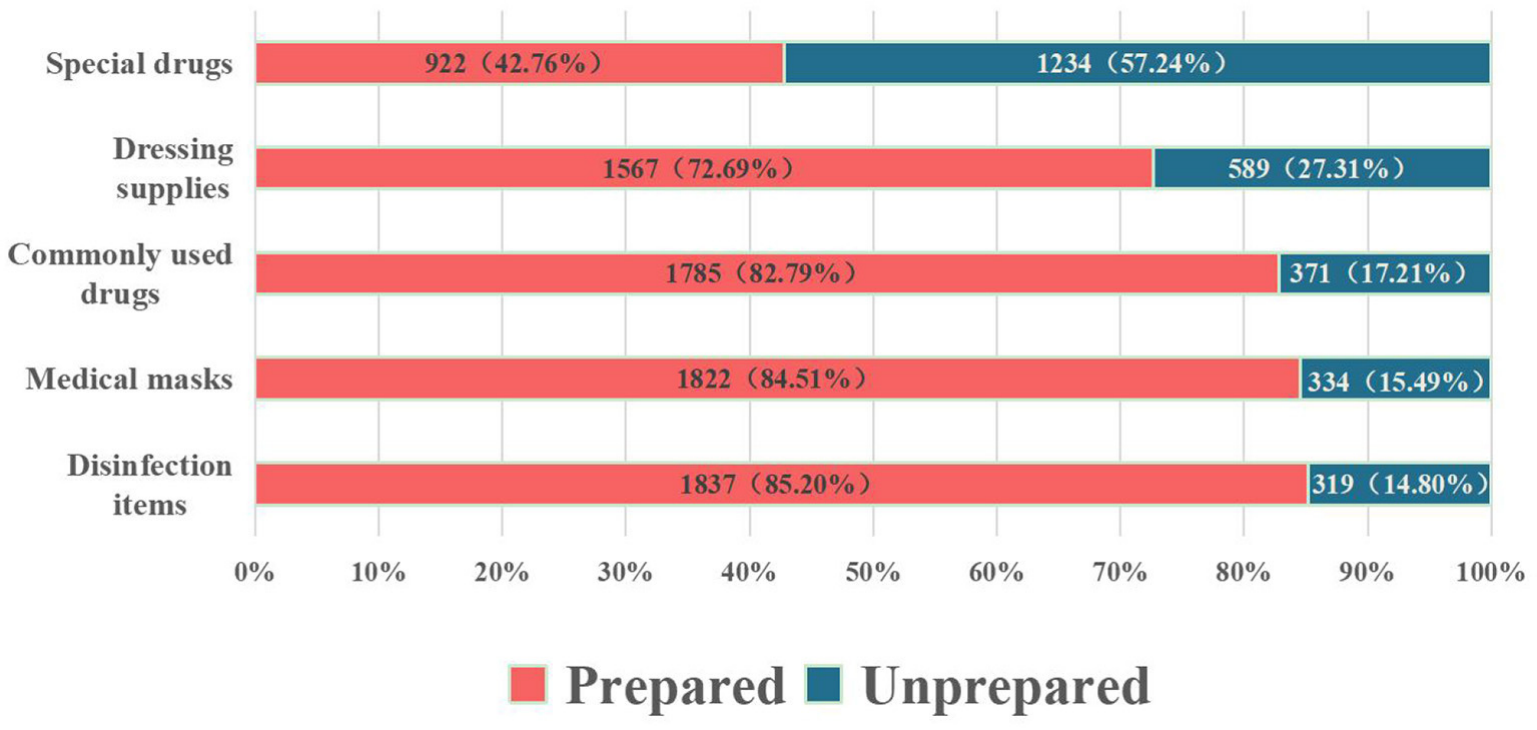
Preparation of items in respondents' home first aid kits.

The multiple-choice question *What emergency supplies are in the home first-aid kit of your household* was answered by 2,156 people who have prepared the home first-aid kits. The number and percentage of people who selected each option are shown in Figure 4. The most frequently selected option was “disinfection items (such as iodine).” Except for seven persons who chose “other,” the option with the least number of choices was “special drugs (such as Suxiaojiuxin pills—Traditional Chinese medicine, emergency medicine for angina pectoris).” The seven respondents who chose “Other” filled in other types of items stored in the home first-aid kit, including drinking water, plasters, eye drops, pet medicines, first-aid bags, and gas masks.

### Self-efficacy, health literacy, and Big Five personality scores of respondents

The scores of NGSES, HLS-SF12, and BFI-10 of the respondents are shown in Table 2. The median and upper and lower quartiles of the respondents' HLS-SF12 scores were 33.33 (30.56, 37.50), and the scores of the health care subscale, disease prevention subscale and health promotion subscale were similar. Among the five subscales of the BFI-10 of the respondents, agreeableness and conscientiousness scored higher, with a median and interquartile range of 7.00 (6.00, 8.00). The rank sum test showed that the average rank of self-efficacy, health literacy, health care, disease prevention, health promotion, extroversion, agreeableness, conscientiousness, and openness of those who had not prepared the home first-aid kit were lower than those of those who had prepared. The average rank of neuroticism was higher among those who did not prepare a home first-aid kit (*P* < 0.05).

**Table 2:** The self-efficacy, health literacy, Big Five personality scores, and rank sum test results^a^.

**Scales**	**Items**	**Score range**	**P50 (P25, P75)**	**People who have prepared home first-aid kit**		**People who have not prepared home first-aid kit**		***Z***	***P*-values**
				**P50 (P25, P75)**	**Average rank**	**P50 (P25, P75)**	**Average rank**		
The New General Self-Efficacy Scale (NGSES)	8	8–40	29.00 (24.00, 32.00)	29.00 (24.00, 32.00)	5,085.20	31.00 (25.00, 32.00)	4,548.71	8.186	<0.001
The Short-Form Health Literacy Instrument (HLS-SF12)	12	0–50	33.33 (30.56, 37.50)	33.33 (30.56, 36.11)	5,179.11	33.33 (31.94, 40.28)	4,520.54	10.110	<0.001
The health care subscale	4	0–50	33.33 (29.17, 37.50)	33.33 (29.17, 37.50)	5,145.02	33.33 (33.33, 41.67)	4,530.77	9.679	<0.001
The disease prevention subscale	4	0–50	33.33 (29.17, 37.50)	33.33 (29.17, 37.50)	5,081.64	33.33 (33.33, 41.67)	4,549.78	8.479	<0.001
The health promotion subscale	4	0–50	33.33 (33.33, 37.50)	33.33 (29.17, 37.50)	5,054.68	33.33 (33.33, 41.67)	4,557.87	7.993	<0.001
The 10-item short version of the Big Five Inventory (BFI-10)	10								
Extroversion	2	2–10	6.00 (5.00, 7.00)	6.00 (5.00, 7.00)	5,168.25	6.00 (5.00, 7.00)	4,523.80	4.801	<0.001
Agreeableness	2	2–10	7.00 (6.00, 8.00)	7.00 (6.00, 8.00)	4,838.68	7.00 (6.00, 7.00)	4,622.66	3.348	0.001
Conscientiousness	2	2–10	7.00 (6.00, 8.00)	7.00 (6.00, 8.00)	4,792.46	7.00 (6.00, 8.00)	4,636.52	2.416	0.016
Neuroticism	2	2–10	6.00 (5.00, 6.00)	6.00 (5.00, 6.00)	4,477.54	6.00 (6.00, 8.00)	4,730.98	−3.981	<0.001
Openness	2	2–10	6.00 (6.00, 7.00)	6.00 (6.00, 7.00)	4,909.40	6.00 (6.00, 8.00)	4,601.44	10.100	<0.001

a The scores of NGSES, HLS-SF12, and BFI-10 showed a non-normal distribution, and the scores were expressed as P50 (P25, P75).

### Analysis of influencing factors of home first-aid kit preparation of respondents

Multivariate analysis was carried out using binary stepwise logistic regression, with home first-aid kit preparedness as the dependent variable, gender, region, permanent residence, age, highest educational level, per capita monthly income of the family, the main way of bearing medical expenses, and the grouping of NGSES scores, HLS-SF12 subscale scores, and BFI-10 subscale scores were independent variables. The table of coding assignments for each independent variable is shown in Supplementary Table 1. The *P*-value of the Ominbus test was 0.033 for the established model, the −2 log-likelihood value was 9,759.669, and the Hosmer-Lameshaw test *P* was 0.624, which was >0.05, indicating that the model was of good quality. The results of the multivariate binary logistic regression showed that region, permanent residence, educational level, per capita monthly household income, self-efficacy, healthcare subscale in HLS-SF12, openness, and agreeableness in the BFI-10 were significantly associated with whether respondents owned a home first-aid kit (*P* < 0.05), as details in Table 3. The tolerance of each variable was between 0.302 and 0.952, all >0.1, and the VIF value was between 1.051 and 3.315, all <10, which indicated that there was little possibility of collinearity in the respective variables in the model.

**Table 3:** Multifactorial stepwise logistic regression analysis of home first aid kit preparation behavior.

**Variable**	**β**	**SE**	**Wald *χ^2^***	***P*-value**	**OR**	**95% *CI* lower limit**	***95%* CI upper limit**	**Tolerance**	***VIF***
**Region (control group: Eastern China)**									
Central China	0.168	0.061	7.665	0.006	1.183	1.050	1.332	0.878	1.139
Western China	0.164	0.063	6.714	0.010	1.179	1.041	1.335	0.871	1.148
**Permanent residence (control group: rural)**									
Urban	0.382	0.065	34.361	<0.001	1.465	1.289	1.664	0.848	1.179
**Highest educational level (control group: primary school and below)**									
Middle School (including junior high school/high school/secondary school)	0.509	0.114	19.921	<0.001	1.664	1.331	2.081	0.353	2.834
Junior college and undergraduate	0.544	0.112	23.650	<0.001	1.723	1.384	2.145	0.302	3.315
Graduate (including Master's and Ph.D. students)	0.711	0.141	25.408	<0.001	2.036	1.544	2.685	0.575	1.739
**Per capita monthly household income (control group:** **≤3,000 yuan)**									
3,001–6,000	0.252	0.067	14.151	<0.001	1.287	1.128	1.467	0.653	1.531
6,001–9,000	0.390	0.081	23.235	<0.001	1.478	1.261	1.732	0.688	1.453
>9,000	0.495	0.084	34.877	<0.001	1.640	1.392	1.933	0.670	1.492
**Self-efficacy (control group: low scoring group)**									
High scoring group	0.211	0.053	15.633	<0.001	1.235	1.112	1.372	0.893	1.120
**Health care (control group: low scoring group)**									
High scoring group	0.255	0.061	17.440	<0.001	1.290	1.145	1.454	0.875	1.143
**Agreeableness (control group: low scoring group)**									
High scoring group	0.111	0.052	4.510	0.034	1.117	1.009	1.237	0.945	1.058
**Openness (control group: low scoring group)**									
High scoring group	0.338	0.067	25.361	<0.001	1.403	1.230	1.600	0.952	1.051

## Discussion

### Home first-aid kit preparation rate and material classification

According to the research results, it is found that the preparation rate of family first aid kits in the Chinese mainland is less than a quarter, and the preparation of family first aid kits in China residents needs to be improved, which is similar to the results of some previous studies in China (33–35). According to a survey conducted by Liu et al. in 2019 (33), only 13.6% of elderly people in Zhengzhou city prepared home first-aid kits. In 2021, Shen et al. (34) investigated the emergency preparedness behavior of residents in Nantong City, Jiangsu Province, and found that only 24.09% of respondents had emergency supplies at home. In 2021, Ning et al. investigated the current status of emergency preparedness behavior among more than 1,700 respondents in Guangdong, Heilongjiang, and Sichuan provinces, and found that the emergency preparation rate of respondents was only 6.59% (35). In addition, some studies have been conducted in other countries to investigate emergency preparedness behavior (36–38). Tomio et al. (36) investigated the emergency preparedness behaviors of Japanese households for earthquake disasters, and the results showed that only 11% of households had a home first-aid kit. Ferguson et al. (37) surveyed the household preparation behaviors in Virginia, USA, where 7.4% of the respondents felt their families were well prepared and 14.4% claimed they were unprepared. Killian et al. (38) explored the emergency preparedness behavior of people over 50 years old in the U.S. and found that the elderly generally do not prepare emergency items such as home first-aid kits. In summary, the preparation of emergency supplies such as home first-aid kits in China and even around the world still needs to be strengthened.

Among the respondents who had prepared a home first-aid kit, the items in the kit were as follows: disinfection items (iodophor, etc.), medical masks, commonly used drugs (anti-inflammatory drugs, painkillers, antipyretics, etc.), dressing supplies (gauze, bandages, etc.), and special drugs (such as Suxiaojiuxin pills and other first-aid drugs). In 2020, the Ministry of Emergency Management of the People's Republic of China issued the “Recommended List of Household Emergency Supplies” (39). The list of supplies includes commonly used drugs (over-the-counter drugs for anti-infection, anti-cold, anti-diarrhea, etc), medical materials (wound dressing such as band-aids, gauze bandages, etc.), iodophor, and cotton swab (for wound treatment and disinfection). In 2022, the Emergency Management Bureau of Haikou City, Hainan Province, issued a “Recommended List of Household Emergency Supplies in Haikou City.” The recommended inventory includes, in addition to the above emergency supplies, medical surgical masks/protective gloves for the prevention of infectious diseases such as COVID-19 (40).

Among the respondents who prepared home first-aid kits, a total of 1,298 (60.20%) respondents prepared disinfection supplies, dressing supplies, and commonly used drugs at the same time. The items prepared by these respondents in their home first-aid kits were consistent with those specified in the “Recommended List of Home Emergency Supplies.” Medical masks can block the spread of harmful gases, odors, droplets, viruses, and other substances, especially when medical supplies are in short supply due to large-scale disasters, and infectious diseases are prone to occur. Therefore, medical masks should be equipped with family first aid kits. In addition, due to the normalization of COVID-19 epidemic prevention and control during the investigation period, masks have become an important tool to prevent the spread of the COVID-19 virus, so many family first-aid kits (84.51%) also are equipped with masks.

### Related factors of home first-aid kit preparation behavior

#### Demographic characteristics

The chi-square test revealed that permanent residence, age, highest education level, per capita monthly household income, and main way of bearing medical expenses were factors associated with home first-aid kit preparation. Multi-factor binary logistic regression analysis of the factors associated with home first-aid kit preparation revealed that permanent residence, location, highest literacy level, per capita monthly household income, and main way of bearing medical expenses were the related factors of home first-aid kit preparation behavior. In terms of permanent residence, urban residents were more likely to have a home first-aid kit than rural residents. Ning et al. (41) explored the emergency preparedness behavior of urban and rural residents in Shaanxi Province for disaster events and found that urban residents were more inclined to stockpile emergency supplies than rural residents, which is consistent with the findings of this study. There are more emergency-related awareness campaigns in urban areas and relatively fewer in rural areas. This is one of the reasons for the gap in disaster preparedness knowledge, attitudes, and behaviors between rural and urban residents (41).

Among the factors of education level, respondents with secondary education or above are more likely to prepare home first-aid kits than those with primary education or below. One study found that respondents with secondary or higher education were 5.02 times more likely to stockpile emergency supplies than those who were illiterate or semi-literate (41). Individuals with higher education have greater access to information, greater crisis awareness, and higher emergency response knowledge and skills (42). Therefore, they are more likely to prepare emergency supplies such as home first-aid kits, in case of an emergency. Among the factors of monthly household income per capita, respondents with higher monthly household income per capita were more inclined to prepare home first-aid kits. The study by Chen et al. also found that monthly household income was one of the factors influencing the incidence of emergency supplies preparedness behaviors among residents, and the higher the monthly household income, the more aware residents were of the need to protect their self-life and property safety (42). Killian et al. (38) found that participation in disaster or emergency preparedness programs was positively, but only slightly, associated with household income (OR = 1.03, *P* < 0.001).

Among the “region” factors, the univariate analysis showed that “region” was not statistically significant, while the multivariate analysis showed that “region” was associated with home first aid kit preparation behavior. The correlation between “region” and home first aid kit preparation behavior was found in the multivariate analysis. The reason for this is the association between the “region” factor and other confounding factors. In the single-factor analysis, the true effect of “region” was masked by the effect of other confounders. After eliminating the effect of other factors through multivariate analysis and testing for covariance, it was found that “region” had an independent effect on family first aid kit preparation behavior. According to the results of the multifactorial analysis, the respondent in central and western China was more likely to prepare home first-aid kits than those in eastern China. This is due to the eastern region has an overall more developed economy and more medical resources compared to the central and western regions, making it convenient for residents to obtain first aid and medication on time (43). In addition, the western region is mostly mountainous or plateau areas with a high risk of earthquakes, mudslides, flash floods, and other geological disasters. Especially Xinjiang, Tibet, Qinghai, and other regions are vast and sparsely populated, and relatively economically and medically backward (44, 45). Therefore, residents in these regions prefer to prepare first-aid kits for emergencies. In terms of medical expenses, compared with self-pay respondents, respondents with resident medical insurance were more likely to prepare home first-aid kits. Differences in the way medical expenses were paid represent differences in the level of medical insurance coverage for the respondents. Those who self-pay for medical expenses bear a larger proportion of medical expenses than those with residents' medical insurance, and their economic income is relatively low (46). Therefore, those who self-paid for their medical expenses, are less likely to prepare a home first-aid kit to prevent emergencies.

#### Effect of self-efficacy on home first-aid kit preparation behavior

Self-efficacy is closely related to a person's confidence in his ability to make effective life-and-death decisions in an emergency (47). In addition, studies have shown that self-efficacy is an important facilitator of health-related intentions and behaviors (48). Self-efficacy is a common facilitator for the public to prepare emergency supplies, develop escape plans, and participate in training and drills. In the present study, we concluded that respondents' self-efficacy was one of the related factors for home first-aid kits preparation behavior. The high self-efficacy subgroup was more likely to prepare home first-aid kits than the low self-efficacy subgroup. American scholars had found that the higher the level of self-efficacy of family members, the more likely they are to adopt emergency preparedness behavior (49). In a study of disaster preparedness in rural families with children requiring special care, Hamann et al. found that self-efficacy had a significant positive contribution to individual emergency preparedness (10). Ryan et al. (50) showed that individuals with low self-efficacy may not engage in emergency preparedness behavior even when they perceive serious risks.

#### Effects of health literacy and Big Five personality on home first-aid kit preparation behavior

In the field of health promotion, health literacy refers to the ability to regularly update one's knowledge of health determinants in social and physical environments, deduce their meanings, explain and evaluate information about health determinants in social and physical environments, and make informed decisions about health determinants in social and physical environments, while participating in joint actions (51). In this study, we found that the health promotion dimension of health literacy was positively associated with respondents' home first-aid kits preparation behavior, with higher scores on the health promotion dimension being associated with a higher likelihood of preparing home first-aid kits. The reason for this may be that respondents with high health promotion scores were more likely to be influenced by the social or physical environment and to take positive health actions. In a case study of disaster health literacy in Dutch, disaster health literacy was found to be critical to public emergency preparedness (52).

Personality precedes any particular attitude or behavior in terms of causation and is an important determinant of various attitudes and behaviors (53). When the outside world provides news or knowledge about disasters, emergency preparedness, etc., people with certain personality traits may receive the information and actively act, such as preparing a home first aid kit. In this study, we found that openness and agreeableness of the Big Five personality were correlated with first-aid kit preparation behavior, and respondents with high openness and agreeableness would be more likely to prepare a home first-aid kit. In a study examining the public's personality and stockpiling behavior during a neocon pneumonia emergency, individuals with high agreeableness and openness tended to stockpile supplies during the emergency. Also, it has been found that people with a pleasant nature are more compliant with precautionary measures (54), strictly follow socially expected safety rules, and try to support and protect others (55). In the present study, preparing a home first aid kit was a precautionary measure to prevent sudden emergencies and facilitate the protection of oneself and others in case of emergency. Therefore, desirability respondents would have higher compliance and be more likely to practice emergency prevention measures such as preparing a first-aid kit as recommended by the government and other authorities. Personality traits may influence health literacy (56), Mai et al. (57) explored the relationship between Big Five personality and health literacy and found that openness had the highest correlation with health literacy. In this study, the high openness group of Big Five personality was more willing to prepare a home first-aid kit than the group with a low degree of openness. We hypothesize that there is a correlation between openness and health literacy, and health literacy has a positive impact on emergency preparedness behavior. Therefore, there is a correlation between openness and home first-aid kit preparation behavior. However, the above explanation is only our speculation, and the mechanism of the influence of Big Five personality on home first-aid kit preparation behavior needs to be studied in depth subsequently.

### Suggestions

As the “first responders” to emergencies and natural disasters, emergency preparedness and self-rescue are mandatory courses for residents. Several studies have found that emergency literacy education and training have an impact on residents' emergency preparedness level and that emergency education and training can enhance residents' risk awareness and improve emergency cognition, thus improving their emergency preparedness behaviors (58, 59). This suggests that we should continue to strengthen the public's attention to emergency supplies such as home first-aid kits and improve public emergency literacy.

In terms of international cooperation, relevant departments of the United Nations should strengthen the worldwide emergency science popularization education from the perspective of the interests of all mankind. For example, the United Nations Office for Disaster Risk Reduction (UNISDR) held an international conference on emergency disasters in Japan and proposed the Sendai Framework for Disaster Risk Reduction 2015 to 2030 (60). Countries with poor emergency preparedness should actively learn from countries with the early development of emergency-related work and better emergency preparedness.

At the national level, the state should strengthen the top-level design, and relevant departments of China government should issue laws and regulations on emergency education. Emergency management agencies should implement the main responsibility, strengthen the arrangement of emergency science education, and strive to build a multi-level emergency knowledge education system of government, community and schools.

In addition, we should give full play to the role of the media. The public's demographic sociological characteristics and personality characteristics are one of the related factors of family first aid kit preparation behavior, which indicates that emergency knowledge education should not be “across the board,” and appropriate and targeted educational interventions should be made for people of different ages, different educational levels, different regions and different income levels. People with different personalities have different characteristics, and they have different degrees of understanding and acceptance of news received from the media. When the relevant departments understand the public's knowledge of the popular science epidemic, they can attach different importance to language expression and popular science content in first aid popular science according to different personality characteristics. For example, people with higher openness will be more curious about things and more receptive to new things. When publicizing the first aid knowledge of popular science, the media can use more novel ways to attract this kind of people at the beginning of popular science education, and then guide readers to understand and learn emergency preparedness knowledge in depth. People with high agreeableness, are more willing to make choices that are beneficial to others and society. Therefore, in the media popularization of first aid knowledge, we can focus on learning emergency knowledge and the benefits of preparing family first-aid kits to attract the attention and recognition of this kind of reader.

Through multi-channel and all-round public emergency education, we should pay attention to the effect of popularization of emergency knowledge, and effectively improve the public's mastery of emergency knowledge and skills, self-efficacy, and emergency attitude, to enhance our residents' emergency literacy and promote more public to prepare emergency materials such as family first aid kits.

### Strengths and limitations

The strengths of this study are as follows. First, this study used a scientific sampling method to investigate the nationwide public perceptions of home first-aid kit preparation and the influencing factors for the first time, and obtained nearly 10,000 representative data. In addition, this study analyzed residents' home first-aid kit preparation behavior by combining self-efficacy, Big Five personality, and health literacy theories, expanding the scope and value of the application of self-efficacy and health literacy theories.

There are several limitations of this study. First, the population base in China is large and our sample data were not fully representative of the actual situation across China. Second, this study used a cross-sectional design that does not allow for causal inference. Third, the data were based entirely on self-report questionnaires, which may be subject to social expectations, self-report errors, and poor memory. Finally, we used the most recent data available to us (2021), however, the preparedness of residents' home first-aid kits and the important factors considered may change further in subsequent years and need to be explored on an ongoing basis.

## Conclusion

Less than a quarter of Chinese residents prepared home first-aid kits, and the supplies among those who prepared home first-aid kits are disinfection supplies, medical masks, commonly used drugs, dressing supplies, and special drugs in order. Demographic factors, self-efficacy, health literacy, and openness in the Big Five personality are related factors influencing the public's home first-aid kit preparation behavior. A multi-layered education system of emergency literacy should be built for government, community, and school, and multi-channel and all-round public emergency education should be carried out to effectively improve the public's self-efficacy and emergency literacy, and eventually improve the residents' attention to emergency supplies such as home first-aid kits and the residents' ability to prevent emergencies.

## Data availability statement

The original contributions presented in the study are included in the article/Supplementary material, further inquiries can be directed to the corresponding authors.

## Ethics statement

This study scheme was approved by the Institutional Review Committee of Ji'nan University, Guangzhou, China (JNUKY-2021-018). All the participants fully understood the study and voluntarily signed informed consent forms. The patients/participants provided their written informed consent to participate in this study.

## Author contributions

PG: conceptualization, methodology, writing—original draft preparation, and writing—review and editing. JZ, KL, YN, QL, PX, JL, YY, YD, XL, MY, and XS: writing—original draft preparation, and writing—review and editing. WY: investigation. XH and YW: conceptualization, validation, and supervision. All authors contributed to the article and approved the submitted version.

## Funding

The research for this article is funded by Annual Project of Shaanxi Provincial Social Science Foundation in 2022 (Grand: 2022M090) and the project of Liaoning Social Science Fund Research on Issues Related to the Integration of Ideological and Political Education in Universities, Middle Schools and Primary Schools (Project Approval Number: L19ASZ004).

## Conflict of interest

The authors declare that the research was conducted in the absence of any commercial or financial relationships that could be construed as a potential conflict of interest. The reviewer XB declared a shared affiliation with the author(s) JZ, MY to the handling editor at the time of review.

## Publisher's note

All claims expressed in this article are solely those of the authors and do not necessarily represent those of their affiliated organizations, or those of the publisher, the editors and the reviewers. Any product that may be evaluated in this article, or claim that may be made by its manufacturer, is not guaranteed or endorsed by the publisher.
