# Supplementary material for: The current status and factors related to the preparation of home first-aid kits in China
Source: Front Public Health. 2022 Nov 28;10:1036299. doi: 10.3389/fpubh.2022.1036299 (PMC9742271; doi:10.3389/fpubh.2022.1036299)
Supplement: Supplementary file 1 [file Table_1.DOCX]

**Supplementary Table 1. Variable assignment table for data analysis**

| **Variable** | **Assignment** |
| --- | --- |
| Gender | 0=Female,1=Male |
| Region | 0=Eastern,1=Central,2=Western |
| Permanent residence | 0=Rural,1=Urban |
| Age | 0=19-35,1=36-59,2=≥60 |
| Highest educational level | 0=primary school and below,1=Middle School (including junior high school/high school/secondary school),2=Junior college and undergraduate,3=Graduate (including Master's and Ph.D. students) |
| Per capita monthly household income | 0= ≤3000, 1=3001-6000, 2=6001-9000, 3=＞9000 |
| The main way of bearing medical expenses | 0=Medical insurance for residents (including medical insurance for urban residents and the new rural cooperative,1=Employee medical insurance, 2=Other types(including commercial medical insurance and public medical care),3=Self-paying |
|  |  |
|  |  |
| Self-efficacy | 0=Low scoring group, 1=High scoring group |
| Health care | 0=Low scoring group, 1=High scoring group |
| Disease prevention | 0=Low scoring group, 1=High scoring group |
| Health promotion | 0=Low scoring group, 1=High scoring group |
| Extroversion | 0=Low scoring group, 1=High scoring group |
| Agreeableness | 0=Low scoring group, 1=High scoring group |
| Conscientiousness | 0=Low scoring group, 1=High scoring group |
| Neuroticism | 0=Low scoring group, 1=High scoring group |
| Openness | 0=Low scoring group, 1=High scoring group |
| Preparation of home first-aid kit | 0=Not prepared, 1=Prepared |

Note: The scores of each scale were classified according to the median. Those with scores higher than or equal to the median were classified as a high-scoring group, and those with scores less than the median were classified as a low-scoring group.


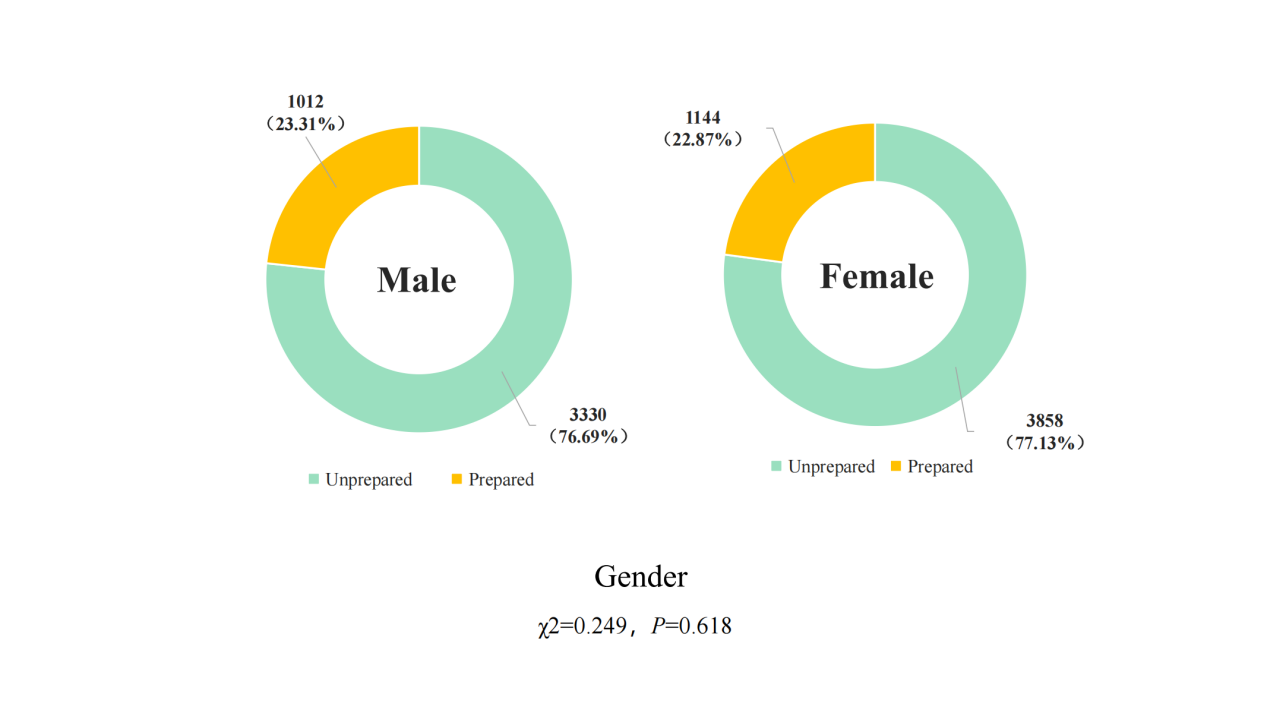


Supplementary Figure 1 Preparation of home first-aid kit by Gender


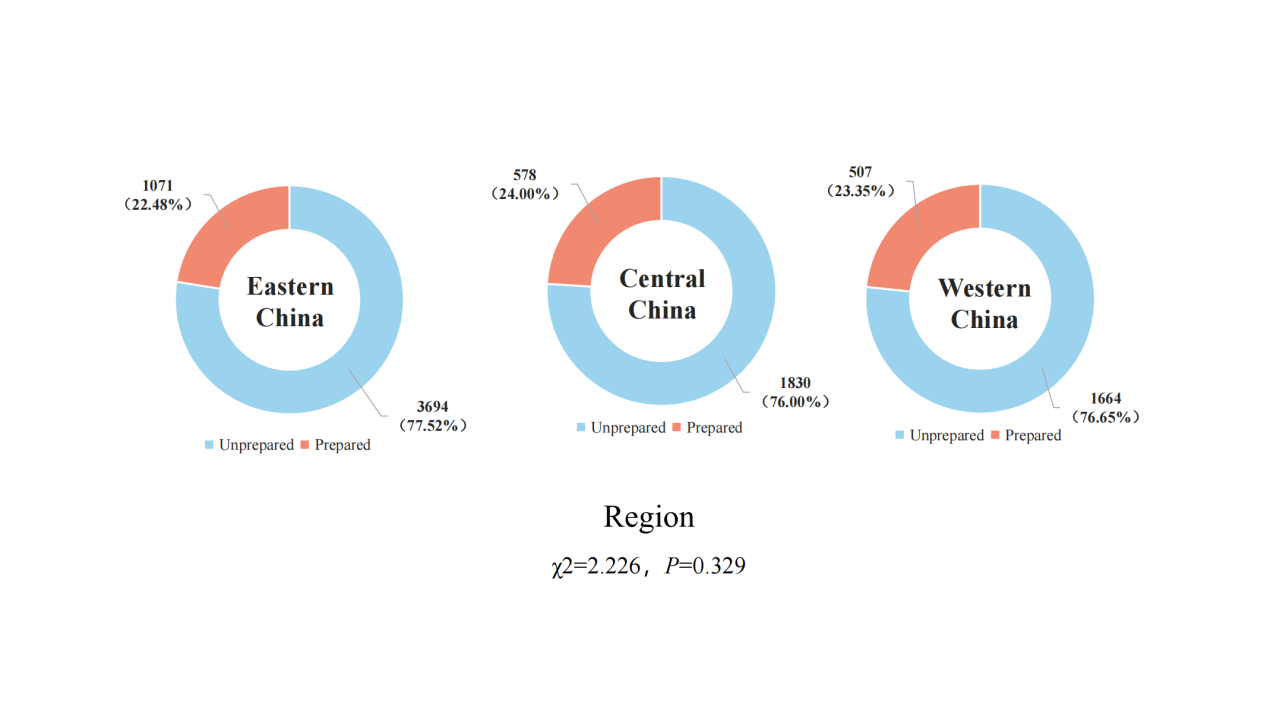


Supplementary Figure 2 Preparation of home first-aid kit by Region
